# A permutation method for detecting trend correlations in rare variant association studies

**DOI:** 10.1017/S0016672319000120

**Published:** 2019-12-13

**Authors:** Lifeng Liu, Pengfei Wang, Jingbo Meng, Lili Chen, Wensheng Zhu, Weijun Ma

**Affiliations:** 1 School of Mathematical Sciences, Heilongjiang University, Harbin 150080, China; 2 Key Laboratory for Applied Statistics of MOE, School of Mathematics and Statistics, Northeast Normal University, Changchun 130024, China

**Keywords:** *γ*-statistic, contingency tables, ordinal variables, rare variants

## Abstract

In recent years, there has been an increasing interest in detecting disease-related rare variants in sequencing studies. Numerous studies have shown that common variants can only explain a small proportion of the phenotypic variance for complex diseases. More and more evidence suggests that some of this missing heritability can be explained by rare variants. Considering the importance of rare variants, researchers have proposed a considerable number of methods for identifying the rare variants associated with complex diseases. Extensive research has been carried out on testing the association between rare variants and dichotomous, continuous or ordinal traits. So far, however, there has been little discussion about the case in which both genotypes and phenotypes are ordinal variables. This paper introduces a method based on the *γ*-statistic, called OV-RV, for examining disease-related rare variants when both genotypes and phenotypes are ordinal. At present, little is known about the asymptotic distribution of the *γ*-statistic when conducting association analyses for rare variants. One advantage of OV-RV is that it provides a robust estimation of the distribution of the *γ*-statistic by employing the permutation approach proposed by Fisher. We also perform extensive simulations to investigate the numerical performance of OV-RV under various model settings. The simulation results reveal that OV-RV is valid and efficient; namely, it controls the type I error approximately at the pre-specified significance level and achieves greater power at the same significance level. We also apply OV-RV for rare variant association studies of diastolic blood pressure.

## 1. Introduction

For the past decade, genome-wide association studies (GWAS) have identified thousands of common variants associated with complex diseases or traits. However, recent evidence suggests that only a small proportion of the phenotypic variance can be explained by common variants (Maher, 2008; Manolio *et al.*, 2009; Eichler *et al.*, 2010; Gibson, 2012). Finding the sources of missing heritability has received considerable critical attention. With the advent of the next-generation of high-throughput DNA sequencing technology, an increasing number of rare variants have been detected. Recent studies have shown that rare variants have the potential to explain part of the missing heritability and may play a key role in the development of complex diseases (Bodmer & Bonilla, 2008; Nelson *et al.*, 2012; Tennessen *et al.*, 2012). Due to the importance of rare variants in sequencing studies, rare variant association analysis has become an increasingly important area in GWAS. To date, a large number of statistical approaches have been proposed for common variant association analysis. However, due to the low mutation rate of rare variants, traditional methods used to test single common variants usually lead to substantial bias and low power in rare variant association analysis (Li & Leal, 2008). To address the above issue, a series of burden tests have been put forward for rare variant association analysis by collapsing a group of rare variants into a specific region. Morgenthaler and Thilly (2007) collapsed the information of the rare variants in a region into a dichotomous variable and provided an approach, called cohort allelic sum test (CAST), for detecting associated rare variants. Some other burden tests for rare variant association studies include the combined multivariate and collapsing method (CMC; Bingshan & Leal, 2008), the sum test (SUM; Pan, 2009) and the weighted sum test (WSS; Madsen & Browning, 2009), among others.

It should be pointed out that all of these burden tests implicitly assume that the effects of rare variants on the phenotype are in the same direction and magnitude (after incorporating known weights), which is obviously unreasonable in GWAS. Recent studies have shown that ignoring the different directions and magnitudes of rare variant effects may lead to loss of testing efficiency (Wu *et al.*, 2011). Hence, there remains a need for developing an efficient rare variant association test, especially when the effects of rare variants on the phenotype are in the different direction and of the same magnitude. In a seminal paper, Wu (2011) proposed a statistical method, termed the ‘sequence kernel association test’ (SKAT), for rare variant association studies. They showed that SKAT allows for different directions and magnitudes of rare variant effects and achieves greater efficiency compared with burden tests. Some extensions of SKAT can be found in the literature (SKAT-O, Lee *et al.*, 2012; HKAT, Lin *et al.*, 2013; W2WK, Broadaway, 2015).

All of the above methods focus on dichotomous or continuous phenotypes. However, in practice, we usually encounter situations in which the genotype or the phenotype is ordinal. For example, it is reasonable to treat the number of risk alleles or the severity of the disease as an ordinal variable. To date, a handful of methods have been proposed for the ordinal phenotype. Diao (2010) developed the variance-components methods for linkage and association analysis of ordinal traits in general pedigrees. Zhou (2016) presented a study that tested the association between rare variants and multiple traits, including ordinal traits or combinations of ordinal traits and other traits. Wang (2017) proposed a method for detecting associations between ordinal traits and rare variants based on the adaptive combination of *p*-values. To date, few studies have investigated the trend correlations between ordinal genotypes and ordinal phenotypes. In this paper, we put forward a method based on the *γ*-statistic, called OV-RV, for detecting disease-related rare variants when both genotypes and phenotypes are ordinal. Due to the extremely low mutation rates for rare variants, the asymptotic distribution of the *γ*-statistic is no longer the normal distribution derived by Goodman (1963). Instead of deriving the asymptotic distribution of the *γ*-statistic for sparse contingency tables, we employ an empirical null hypothesis by utilizing the permutation approach proposed by Fisher. We carry out extensive simulations to compare the numerical performance of OV-RV with several existing approaches in a wide range of model settings. The simulation results demonstrate that OV-RV is valid and efficient.

The remainder of this paper is organized as follows: in Section 2, we provide a brief description of the cross-contingency table in categorical data analysis. Then, we introduce a measure called *γ* for detecting the association between two ordinal variables and show that the asymptotic distribution of the *γ*-statistic is no longer applicable in rare variant association studies. To address this issue, a detailed permutation approach is provided. Extensive simulations and a real data analysis are conducted in Section 3. Section 4 contains a discussion of our results and some potential extensions of our approach.

## 2. Method

Suppose there are *n* independent subjects in a population-based study. For each subject *i*, we let *Y*_*i*_ be the phenotype and (*G*_*i*1_, …, *G*_*im*_) be the genotype at the *m* loci, where *G*_*ij*_ is the number of mutations in variant *j* for subject *i*. In general, *G*_*ij*_ ∈ {0, 1, 2}. The genetic score of the genotype for subject *i* is defined as

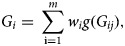

where *w*_*i*_ is a weight and *g*( · ) is a link function. In practice, the selection of the weight and the use of the link function can be of various types as long as they are justified. For example, one can choose the weight utilized in Madsen and Browning (2009) to ensure that all variants in a group contribute equally. In this paper, we choose the weight *w*_*i*_ = 1 and the link function 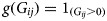, where 1_(.)_ is an indicator function. At last, according to the genetic score, the genotype levels are sorted from small to large. Correspondingly, the phenotypes can be sorted from small to large in terms of the degree of the disease. For *i* = 1, …, *n*, let *i* and *j* be the numbers of different *Y*_*i*_ and different *G*_*i*_, respectively. For ease of notation, denote by *Y* the phenotype and denote by *G* the genotype score at the *m* loci. Let 0, 1, …, *I* − 1 and 0, 1, …, *J* − 1 be the levels of *Y* and *G*, respectively.

### 2.1. The cross-contingency table

The cross-contingency table is a tool that can properly display the joint distribution of categorical variables and has been widely used in categorical data analysis. In order to express the framework of the *γ*-statistic explicitly, we first provide a brief description of the cross-contingency table for rare variant association studies. The cross-contingency table of the genotype level at *m* loci by the phenotype level is listed in Table 1, where *x*_*ij*_ is the number of subjects that occurs in the cell in row *i* and column *j*. Denote by *π*_*ij*_ the joint probability of (*Y*, *G*) in the cell of row *i* and column *j* and by {*π*_*ij*_}_*I*×*J*_ the joint distribution of (*Y*, *G*).

**Table 1.** Cross-contingency table of genotype at the *m* loci by phenotype.

	Genotype level at the *m* loci			
Phenotype level	0	1	…	*J-1*
0	*x*_11_	*x*_12_	…	*x*_1*J*_
1	*x*_21_	*x*_22_	…	*x*_2*J*_
2	*x*_31_	*x*_32_	…	*x*_3*J*_
⋮	⋮	⋮	⋮	⋮
*I* − 1	*x*_*I*1_	*x*_*I*2_	…	*x*_*IJ*_

### 2.2. The *γ*-statistic

When both *Y* and *G* are ordinal, one would expect to test the monotone trend association between *Y* and *G*, where the monotone trend association refers to *Y* trending to increase to higher levels or trending to decrease to lower levels as the level of *G* increases. Define that a pair of subjects is concordant if there exists a subject that ranks higher on *G* and *Y* simultaneously. Similarly, define that a pair of subjects is discordant if there exists a subject that ranks higher on *G* but ranks lower on *Y*. Consider two independent observations randomly sampled from the joint distribution {*π*_*ij*_}_*I*×*J*_. For this pair of subjects, we can express the probabilities of concordance and discordance as follows:
1
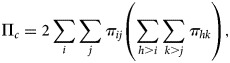

and
2
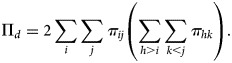

Then, a natural association measure to describe the monotone trend association is the difference Π_*c*_ − Π_*d*_.

Assume that a pair is untied on both *Y* and *G*; in other words, the probability of ties *Y*_*i*_ = *Y*_*j*_ or *G*_*i*_ = *G*_*j*_ is zero. Then, Π_*c*_/(Π_*c*_ + Π_*d*_) and Π_*d*_/(Π_*c*_ + Π_*d*_) are the probabilities of concordance and discordance, respectively. Goodman (1954) suggested utilizing the difference between these probabilities to measure this trend. Specifically, the measure called *γ* is defined as
3
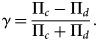


Correspondingly, the sample version is
4
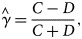

where 
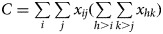
 and 
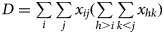
 are the total numbers of concordant pairs and discordant pairs, respectively.

Note that testing *H*_0_: *Y* and *G* are independent can be reduced to testing *H*_0_: *γ* = 0, when both *Y* and *G* are ordinal. Goodman (1963) further derived the asymptotic distribution of the *γ*-statistic under the null hypothesis. However, in rare variant association studies, the asymptotic distribution is no longer applicable. This is because the low mutation rate of rare variants results in most of *x*_*ij*_ being extremely small or even equal to zero, which in turn leads to bias of the asymptotic distribution.

### 2.3. The permutation approach

In this section, we provide a detailed permutation approach for estimating the distribution of the *γ*-statistic in what follows.

Step 1. For *a* = 1, …, *A*, execute the following steps:

- Randomly permute the original phenotype (*Y*_1_, *Y*_2_, …, *Y*_*n*_);
- Generate the new cross-contingency table by matching the permuted phenotype (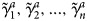) and the genotype (*G*_1_, *G*_2_, …, *G*_*n*_);
- Calculate the *γ*-statistic 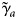 based on the new cross-contingency table.

Step 2. Estimate the distribution of the *γ*-statistic under the null hypothesis:

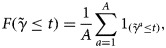

where 1_(.)_ is an indicator function.

## 3. Simulation studies

In this section, we explore the numerical performance of our method (OV-RV) and five existing methods, including CAST (Morgenthaler & Thilly, 2007), SUM (Pan, 2009), WSS (Madsen & Browning, 2009), SKAT (Wu *et al.*, 2011) and SKAT-O (Lee *et al.*, 2012). It is necessary to note that SKAT and SKAT-O cannot be directly used for the situation with ordinal traits. To test the associations when both the trait and the genotype are ordinal variables, one potential adjustment is to dichotomize the ordinal phenotype variables (still named SKAT and SKAT-O) and the alternative is to treat the ordered variables as continuous variables (named SKAT-C and SKAT-O-C). We compare these testing methods in terms of two aspects. First, we determine whether these methods can control the type I error at the prespecified *α* level. Without loss of generality, the prespecified *α* levels are set to be 0·05 and 0·01 in the simulations. Second, we compare the power of these methods at the same significance level. According to the scheme for generating simulated data, the simulations are divided into two cases, including a designed parameter-based simulation and a real genotype-based simulation. The simulation results are based on 1000 replications.

### 3.1. Simulation I

In this simulation, we set the sample size *n* = 500 and consider a region of loci that consists of *m* rare variants. Without loss of generality, *m* is set to be 20 and 40, respectively. We first generate ordinal genotype variables and then use continuous intermediate variables to generate ordinal phenotype variables.

For each locus *j*, let *p*_*j*_ be the minor allele frequencies (MAFs) of the corresponding rare variants. Within each region, we randomly sampled *p*_*j*_ from the uniform distribution *U*(0.001, 0.01). Under the assumptions of the Hardy–Weinberg equilibrium law, the probabilities that the genotype score *G*_*ij*_ has a value of 0, 1 and 2 are (1 − *p*_*j*_)^2^, 2*p*_*j*_(1 − *p*_*j*_) and 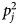, respectively. According to the number of mutant loci in each subject, the genetic scores *G*_*i*_, *i* = 1, …, *n* are classified into *j* ordinal categories, where 
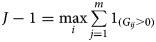
.

To focus on the main points, we select 12 and 18 rare variants from the region of 20 and 40 rare variants as disease-causal variants, respectively. The intermediate variables *T*_*i*_, *i* = 1, …, *n* are generated by the following linear model:

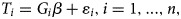

where *ɛ*_*i*_, *i* = 1, …, *n* are independent and 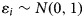, and *β* = *d* · (1,1,0,1,1,0,1,0,0,1,1,0,0,1,1,0,1,1,0,1)^*T*^ if *m* = 20 and *β* = *d* · (1,0,1,1,0,0,1,0,1,0,1,0,0,1,0,0,1,0,1,0,1,1,0,0,1,0,1,0,1,0,0,1,0,0,1,0,1,0,1,0)^*T*^ if *m* = 40. In the following simulation, the values of *d* are set to be 0, 0·2, 0·4, 0·6 and 0·8, respectively. It is clear that *T*_*i*_ and *G*_*i*_ are independent when *d* = 0. Hence, examining the control of type I errors yielded by these testing methods is under the model setting *d* = 0. We use the 20%, 30% and 40% sample percentiles to discretize *T*_*i*_, *i* = 1, …, *n* and generate ordinal phenotype variables *Y*_*i*_, which take values of 0, 1, 2 and 3. The simulation results are exhibited in Tables 2 and 3.

**Table 2.** Estimated type I errors of the eight methods in Simulation I.

*n* = 500	Test	20 rare variants	40 rare variants
*α* = 0.01	OV-RV	0.007	0.008
	SKAT-O-C	0.011	0.005
	SKAT-O	0.011	0.007
	SKAT-C	0.009	0.007
	SKAT	0.009	0.009
	CAST	0.007	0.007
	SUM	0.006	0.011
	WSS	0.009	0.007
*α* = 0.05	OV-RV	0.049	0.049
	SKAT-O-C	0.042	0.046
	SKAT-O	0.038	0.049
	SKAT-C	0.040	0.036
	SKAT	0.043	0.049
	CAST	0.035	0.051
	SUM	0.037	0.047
	WSS	0.033	0.056

**Table 3.** Estimated power results of the eight methods based on the generated genotypes.

*α* level	Number of rare variants	Test	*d* = 0.8	*d* = 0.6	*d* = 0.4	*d* = 0.2
*α* = 0.01	20 (12 causal)	OV-RV	0.761	0.504	0.221	0.070
		SKAT-O-C	0.672	0.400	0.123	0.026
		SKAT-O	0.276	0.151	0.054	0.006
		SKAT-C	0.201	0.058	0.013	0.003
		SKAT	0.012	0.006	0.001	0.001
		CAST	0.392	0.252	0.100	0.023
		SUM	0.403	0.251	0.096	0.023
		WSS	0.306	0.191	0.079	0.015
	40 (18 causal)	OV-RV	0.788	0.559	0.269	0.067
		SKAT-O-C	0.756	0.475	0.179	0.032
		SKAT-O	0.331	0.199	0.072	0.016
		SKAT-C	0.229	0.077	0.022	0.005
		SKAT	0.010	0.009	0.004	0.005
		CAST	0.388	0.241	0.105	0.031
		SUM	0.434	0.276	0.123	0.028
		WSS	0.357	0.215	0.095	0.022
*α* = 0.05	20 (12 causal)	OV-RV	0.914	0.723	0.447	0.160
		SKAT-O-C	0.875	0.665	0.337	0.115
		SKAT-O	0.564	0.393	0.186	0.071
		SKAT-C	0.518	0.268	0.099	0.039
		SKAT	0.063	0.039	0.024	0.019
		CAST	0.634	0.459	0.231	0.105
		SUM	0.684	0.498	0.259	0.125
		WSS	0.627	0.460	0.241	0.108
	40 (18 causal)	OV-RV	0.930	0.776	0.474	0.177
		SKAT-O-C	0.920	0.730	0.416	0.126
		SKAT-O	0.617	0.423	0.238	0.088
		SKAT-C	0.549	0.276	0.084	0.040
		SKAT	0.074	0.040	0.029	0.028
		CAST	0.625	0.444	0.267	0.100
		SUM	0.710	0.548	0.326	0.127
		WSS	0.644	0.484	0.281	0.112

Table 2 presents the empirical sizes of the eight methods at different prespecified significance levels. From the upper part of Table 2, we can observe that all eight methods can control type I errors at the nominal level of approximately 0·01, except for the case in which the empirical type I error of SKAT-O-C is relatively conservative when *m* = 40 and *α* = 0.01. From the lower part of Table 2, similar results are obtained when *α* = 0.05. Although the empirical type I error of WSS is relatively large when *m* = 40 and *α* = 0.05, it is still acceptable. These simulation results confirm the validity of the eight methods in Simulation I.

Table 3 displays the power of the eight methods at different prespecified significance levels and different parameter settings. From Table 3, we can see that the power yielded by the eight methods is decreasing when *d* varies from 0·8 to 0·2. Note that the larger the value of *d*, the stronger the trend association between the ordinal phenotype variable and the ordinal genotype variable. It is easy to interpret the aforementioned simulation results. We can also observe that the power of OV-RV uniformly dominates the other competing methods. This indicates that our OV-RV is more efficient, especially when both the phenotype and the genotype are ordinal.

### 3.2. Simulation II

In this section, we perform simulations to evaluate the numerical performance of OV-RV and its competing methods on more realistic simulated data. In order to simulate data with realistic linkage disequilibrium patterns, we choose the real genotypes of 697 unrelated subjects from Genetic Analysis Workshop 17 (GAW17, http://www.1000genomes.org). Specifically, we select two genes, *TG* and *COL6A3*, as candidate genes. The *TG* gene contains 146 single-nucleotide polymorphisms (SNPs), among which 113 out of these 146 SNPs are rare (MAF <1%), whereas the *COL6A3* gene consists of 187 SNPs, and 143 out of these 187 SNPs are rare. The means of action of these genes have been revealed in several studies (Baker *et al.*, 2005; Maierhaba *et al.*, 2008). For example, Maierhaba (2008) pointed out that the *TG* gene encodes thyroglobulin and may lead to hypothyroidism and autoimmune disorders.

Likewise, for each of these two genes, we randomly selected 20 and 40 rare variants to form a region of loci, respectively. Assume that the effects of rare variants on the phenotype are in the same direction. The rest of the method for generating the ordinal phenotype values is the same as in Simulation I, and we omit the details. The detailed simulation results of the empirical sizes are listed in Tables 4 and 5. To further illustrate the superiority of OV-RV in detecting trend associations, we conduct simulations to compare the power of these methods and list the results in Tables 6 and 7.

**Table 4.** Estimated type I errors of the *TG* gene of the eight methods.

*n* = 697	Test	20 rare variants	40 rare variants
*α* = 0.01	OV-RV	0.012	0.010
	SKAT-O-C	0.008	0.007
	SKAT-O	0.009	0.007
	SKAT-C	0.006	0.011
	SKAT	0.006	0.007
	CAST	0.010	0.005
	SUM	0.013	0.008
	WSS	0.012	0.008
*α* = 0.05	OV-RV	0.048	0.054
	SKAT-O-C	0.037	0.056
	SKAT-O	0.041	0.051
	SKAT-C	0.038	0.050
	SKAT	0.038	0.041
	CAST	0.045	0.050
	SUM	0.053	0.057
	WSS	0.050	0.054

**Table 5.** Estimated type I errors of the *COL6A3* gene of the eight methods.

*n* = 697	Test	20 rare variants	40 rare variants
*α* = 0.01	OV-RV	0.012	0.011
	SKAT-O-C	0.011	0.010
	SKAT-O	0.007	0.015
	SKAT-C	0.012	0.008
	SKAT	0.012	0.012
	CAST	0.011	0.006
	SUM	0.013	0.016
	WSS	0.011	0.011
*α* = 0.05	OV-RV	0.050	0.051
	SKAT-O-C	0.043	0.049
	SKAT-O	0.053	0.044
	SKAT-C	0.044	0.051
	SKAT	0.052	0.047
	CAST	0.037	0.028
	SUM	0.049	0.038
	WSS	0.053	0.044

**Table 6.** Estimated power results of the *TG* gene of the six methods.

*α* level	Number of rare variants	Test	*d* = 0.8	*d* = 0.6	*d* = 0.4	*d* = 0.2
*α* = 0.01	20 (12 causal)	OV-RV	0.859	0.590	0.266	0.044
		SKAT-O-C	0.687	0.400	0.125	0.016
		SKAT-O	0.208	0.108	0.029	0.003
		CAST	0.554	0.340	0.135	0.025
		SUM	0.335	0.176	0.057	0.006
		WSS	0.340	0.175	0.063	0.003
	40 (18 causal)	OV-RV	0.933	0.742	0.345	0.096
		SKAT-O-C	0.878	0.577	0.215	0.040
		SKAT-O	0.405	0.230	0.068	0.019
		CAST	0.605	0.368	0.144	0.047
		SUM	0.653	0.406	0.171	0.048
		WSS	0.480	0.279	0.110	0.041
*α* = 0.05	20 (12 causal)	OV-RV	0.962	0.808	0.498	0.148
		SKAT-O-C	0.903	0.677	0.336	0.083
		SKAT-O	0.545	0.333	0.139	0.038
		CAST	0.780	0.512	0.250	0.078
		SUM	0.700	0.489	0.223	0.065
		WSS	0.719	0.505	0.246	0.080
	40 (18 causal)	OV-RV	0.984	0.888	0.596	0.213
		SKAT-O-C	0.971	0.835	0.486	0.156
		SKAT-O	0.767	0.522	0.264	0.090
		CAST	0.832	0.622	0.340	0.125
		SUM	0.852	0.650	0.348	0.127
		WSS	0.811	0.617	0.338	0.125

**Table 7.** Estimated power results of the *COL6A3* gene of the six methods.

*α* level	Number of rare variants	Test	*d* = 0.8	*d* = 0.6	*d* = 0.4	*d* = 0.2
*α* = 0.01	20 (12 causal)	OV-RV	0.805	0.543	0.235	0.044
		SKAT-O-C	0.644	0.352	0.108	0.014
		SKAT-O	0.177	0.093	0.028	0.008
		CAST	0.429	0.270	0.107	0.035
		SUM	0.425	0.263	0.099	0.033
		WSS	0.249	0.135	0.053	0.015
	40 (18 causal)	OV-RV	0.906	0.670	0.320	0.061
		SKAT-O-C	0.819	0.519	0.180	0.024
		SKAT-O	0.320	0.155	0.048	0.009
		CAST	0.577	0.316	0.151	0.040
		SUM	0.515	0.273	0.117	0.025
		WSS	0.380	0.192	0.074	0.023
*α* = 0.05	20 (12 causal)	OV-RV	0.944	0.770	0.445	0.165
		SKAT-O-C	0.893	0.669	0.329	0.105
		SKAT-O	0.525	0.348	0.186	0.066
		CAST	0.627	0.419	0.220	0.083
		SUM	0.625	0.403	0.209	0.070
		WSS	0.628	0.426	0.229	0.087
	40 (18 causal)	OV-RV	0.976	0.862	0.578	0.176
		SKAT-O-C	0.954	0.807	0.459	0.112
		SKAT-O	0.670	0.425	0.199	0.060
		CAST	0.715	0.488	0.245	0.070
		SUM	0.780	0.575	0.302	0.092
		WSS	0.736	0.533	0.296	0.103

Table 4 displays the empirical type I errors of the eight methods for the *TG* gene. The upper part of Table 4 presents the results with the significance level *α* = 0.01, whereas the lower part of Table 4 lists the results with *α* = 0.05. From Table 4, it is apparent that OV-RV controls the empirical type I errors properly at the different significance levels. We can also see that SKAT is always conservative for the *TG* gene. This phenomenon may be largely due to the improper dichotomization for SKAT. Table 5 presents the empirical type I errors of the eight methods for the *COL6A3* gene. It can be observed that the simulation results are almost wholly consistent with those in Table 4. Although the empirical type I error yielded by OV-RV is a little aggressive when *α* = 0.01 and *m* = 20, it is still acceptable. Overall, these results further indicate that the distribution of the *γ*-statistic under the null hypothesis can be properly estimated by exploiting the permutation method appropriately.

Tables 6 and 7 exhibit the simulation results of the power comparisons of the six methods for the *TG* gene and the *COL6A3* gene in Simulation II, respectively. Due to the extremely low power of SKAT and SKAT-C, we do not list their simulation results in these tables. It is clear that OV-RV shows a significant improvement in power compared with the other five methods at all model settings. By employing the *γ*-statistic, OV-RV can achieve greater efficiency for detecting the trend associations. Similarly, we can also conclude that the power of these methods is increasing in the parameter *d*. We can also determine that the power when *m* = 40 is uniformly larger than the corresponding power when *m* = 20. Under the assumption that the effects of rare variants on the phenotype are in the same direction, a larger number of causal rare variants implies a stronger trend association with the same value of *d*. Hence, it is easy to interpret the results.

We carry out additional simulation studies for OV-RV in testing the effects with different directions. The detailed simulation results are displayed in Additional File 1. When a small proportion of effect directions are different, the simulation results are almost wholly consistent with those in the previous simulations. However, the power of OV-RV decreases as the proportion of effects in different directions increases. This indicates that OV-RV is conservative when a large proportion of effects are of different directions. A more powerful selection of the genetic score may shed light on how to extend OV-RV to these situations, and we plan to pursue this approach in our further research.

## 4. Application to the detection of disease-related genes

In this section, we further apply OV-RV for the detection of disease-related genes on a real dataset called Genetic Analysis Workshop 19 (GAW19). The GAW19 dataset contains whole genome and exome sequences for odd chromosomes, gene expression measures, systolic blood pressure and diastolic blood pressure (DBP), as well as related covariates in 20 large families and 1943 unrelated individuals. Here, we focus on the 1943 unrelated individuals provided by GAW19 and consider the DBP phenotype. A series of procedures for data pre-processing are performed before carrying out association studies. We eliminate individuals who have missing phenotypes, and a total of 1851 individuals are left for analysis. In addition, we complete the missing genotype by a random sample based on the MAF.

DBP is measured in millimetres of mercury (mmHg) when the heart is at rest between beats. It has been reported that genes *EBF1* and *NPR3* on chromosome 5, as well as gene *TMEM133* on chromosome 11, are associated with DBP (Sun *et al.*, 2016). We apply our proposed OV-RV to test associations between these genes and DBP. From the hg19 reference (see https://www.cog-genomics.org/static/bin/plink/glist-hg19), we can obtain the gene starts and gene ends of these three genes. For each gene, genotypes are generated by selecting rare variant loci with MAF <5%. The significance level is set to be 0·05. The phenotypes are divided into four levels in terms of DBP. To be specific, phenotypes with DBP <60, 60 ⩽ DBP <80, 80 ⩽ DBP <90 and DBP ⩾ 90 correspond to levels 0, 1, 2 and 3, respectively. Due to the poor performance of the CAST, SUM and WSS methods in simulations, we only compare the performance of the remaining five methods. Detailed results are shown in Table 8. It is clear that the *p*-values yielded by OV-RV are uniformly smaller than those of the competing methods. We can also see that OV-RV identifies all three DBP-related genes, whereas the other competing methods identify at most one related gene. This indicates that OV-RV is more efficient at detecting disease-related genes.

**Table 8.** The Genetic Analysis Workshop 19 (GAW19) data shown as a list of genes associated with diastolic blood pressure.

Chromosome	Gene	Method				
		OV-RV	SKAT-O-C	SKAT-O	SKAT-C	SKAT
5	*EBF1*	0.003	0.069	0.289	0.088	0.558
5	*NPR3*	0.037	0.214	0.266	0.340	0.160
11	*TMEM133*	0.044	0.048	0.401	0.048	0.359

## 5. Discussion

In this paper, we propose a novel method, called OV-RV, for the detection of the trend associations between ordinal genotypes and ordinal phenotypes. The *γ*-statistic has been successfully applied to the field of searching for the trend associations. However, the asymptotic distribution of the *γ*-statistic derived by Goodman (1963) is no longer valid for rare variant associations. Instead of using the asymptotic distribution directly in rare variant associations, we utilize the permutation method to estimate the distribution of the *γ*-statistic under the null hypothesis. Both the designed parameter-based simulation and the real genotype-based simulation illustrate that OV-RV is valid and more efficient compared with its competitors. A real data analysis on the GAW19 dataset shows that OV-RV achieves greater efficiency and can detect more disease-related genes.

Our OV-RV can also be extended in several ways. First, it has been shown that different diseases or traits usually share similar genetic mechanisms. Conducting an integrative association analysis of several traits can significantly improve testing efficiency. Hence, it is desirable to develop a method for testing associations between ordinal genotypes and multiple ordinal phenotypes. Second, as illustrated in simulations, the power of OV-RV decreases as the proportion of effects in different directions increases. This means that OV-RV is conservative when a large proportion of effects are of different directions. It would be of interest to obtain a more powerful genetic score for extending OV-RV to these situations. Third, the permutation method brings large computation costs when there is a large number of rare variants. Recently, algebraic statistics has been successfully applied in testing independence from the sparse contingency table. It may give rise to a novel method for testing trend associations from the sparse contingency table.

## Supplementary material

For supplementary material accompanying this paper visit https://doi.org/10.1017/S0016672319000120. click here to view supplementary material

## References

[ref1] Baker N. L., Mörgelin M., Peat R. (2005). Dominant collagen VI mutations are a common cause of Ullrich congenital muscular dystrophy. Human Molecular Genetics 14, 279–293. 15563506 10.1093/hmg/ddi025

[ref2] Bingshan L. and Leal S. M. (2008). Methods for detecting associations with rare variants for common diseases: application to analysis of sequence data. American Journal of Human Genetics 83, 311–321. 18691683 10.1016/j.ajhg.2008.06.024 PMC2842185

[ref3] Bodmer W. and Bonilla C. (2008). Common and rare variants in multifactorial susceptibility to common diseases. Nature Genetics 40, 695–701. 18509313 10.1038/ng.f.136 PMC2527050

[ref4] Broadaway K. A. (2015). Kernel approach for modeling interaction effects in genetic association studies of complex quantitative traits. Genetic Epidemiology 39, 366–375. 25885490 10.1002/gepi.21901 PMC4469530

[ref5] Diao G. and Lin D. Y. (2010). Variance-components methods for linkage and association analysis of ordinal traits in general pedigrees. Genetic Epidemiology 34, 232–237. 19918762 10.1002/gepi.20453 PMC3003595

[ref6] Eichler E. E., Flint J., Gibson G. (2010). Missing heritability and strategies for finding the underlying causes of complex disease. Nature Reviews Genetics 11, 446–450. 10.1038/nrg2809 PMC2942068 20479774

[ref7] Gibson G. (2012). Rare and common variants: twenty arguments. Nature Reviews Genetics 13, 135–145. 10.1038/nrg3118 PMC4408201 22251874

[ref8] Goodman L. A. and Kruskal W. H. (1954). Measures of association for cross classifications. Journal of the American Statistical Association 49, 732–746.

[ref9] Goodman L. A. and Kruskal W. H. (1963). Measures of association for cross classifications. III: Approximate sampling theory. Journal of the American Statistical Association 58, 310–364.

[ref10] Lee S., Wu M. and Lin X. (2012). Optimal tests for rare variant effects in sequencing association studies. Biostatistics 4, 762–775. 10.1093/biostatistics/kxs014 PMC3440237 22699862

[ref11] Li B. and Leal S. M. (2008). Methods for detecting associations with rare variants for common diseases: application to analysis of sequence data. American Journal of Human Genetics 83, 311–321. 18691683 10.1016/j.ajhg.2008.06.024 PMC2842185

[ref12] Lin W. Y., Yi N., Lou X. Y. (2013). Haplotype kernel association test as a powerful method to identify chromosomal regions harboring uncommon causal variants. Genetic Epidemiology 37, 560–570. 23740760 10.1002/gepi.21740 PMC4116485

[ref13] Madsen B. E. and Browning S. R. (2009). A groupwise association test for rare mutations using a weighted sum statistic. PLoS Genetics 5, e1000384. 19214210 10.1371/journal.pgen.1000384 PMC2633048

[ref14] Maher B. (2008). Personal genomes: the case of the missing heritability. Nature 456, 18–21. 18987709 10.1038/456018a

[ref15] Maierhaba M., Zhang J. A., Yu Z. Y. (2008). Association of the thyroglobulin gene polymorphism with autoimmune thyroid disease in Chinese population. Endocrine 33, 294–299. 19034705 10.1007/s12020-008-9082-x

[ref16] Manolio T. A., Collins F. S., Cox N. J. (2009). Finding the missing heritability of complex diseases. Nature 461, 747–753. 19812666 10.1038/nature08494 PMC2831613

[ref17] Morgenthaler S. and Thilly W. G. (2007). A strategy to discover genes that carry multi-allelic or mono-allelic risk for common diseases: a cohort allelic sums test (CAST). Mutation Research 615, 28–56. 17101154 10.1016/j.mrfmmm.2006.09.003

[ref18] Nelson M. R., Wegmann D., Ehm M. G., (2012). An abundance of rare functional variants in 202 drug target genes sequenced in 14,002 people. Science 337, 100–104. 22604722 10.1126/science.1217876 PMC4319976

[ref19] Pan W. (2009). Asymptotic tests of association with multiple SNP in linkage disequilibrium. Genetic Epidemiology 5, e1000384. 10.1002/gepi.20402 PMC2732754 19170135

[ref20] Sun J., Bhatnagar S. R., Oualkacha K., Ciampi A. and Greenwood C. M. T. (2016). Joint analysis of multiple blood pressure phenotypes in GAW19 data by using a multivariate rare-variant association test. BMC Proceedings 10, 309–313. 27980654 10.1186/s12919-016-0048-3 PMC5133485

[ref21] Tennessen J. A., Bigham A. W., O'Connor T. D. (2012). Evolution and functional impact of rare coding variation from deep sequencing of human exomes. Science 337, 64–69. 22604720 10.1126/science.1219240 PMC3708544

[ref22] Wang M., Ma W. and Zhou Y. (2017). Association detection between ordinal trait and rare variants based on adaptive combination of p values. Journal of Human Genetics 63, 37–45. 29215083 10.1038/s10038-017-0354-2

[ref23] Wu M., Lee S., Cai T., Li Y., Boehnke M. and Lin X. (2011). Rare-variant association testing for sequencing data with the sequence kernel association test. American Journal of Human Genetics 89, 82–93. 21737059 10.1016/j.ajhg.2011.05.029 PMC3135811

[ref24] Zhou Y., Cheng Y., Zhu W. and Zhou Q. (2016). A nonparametric method to test for associations between rare variants and multiple traits. Genetics Research 98, e1. 27159928 10.1017/S0016672315000269 PMC6865163

