# Supplementary material for: A permutation method for detecting trend correlations in rare variant association studies
Source: Genet Res (Camb). 2019 Dec 13;101:e13. doi: 10.1017/S0016672319000120 (PMC7044977; doi:10.1017/S0016672319000120)
Supplement: Supplementary file 1 [file S0016672319000120sup001.doc]

1. Simulation studies for OV-RV in testing the effects with different directions. Pr stands for the causal variants effect with different directions.

**Table 1**: Power estimation of the COL6A3 gene in the presence of reverse causal variants

|  |  |  |  | $d=0.8$ |  |
| --- | --- | --- | --- | --- | --- |
| Level $\alpha$ | Number of RVs | Text | Pr=1/6 | Pr=1/3 | Pr=1/2 |
| $\alpha=0.01$ | 20 (12 causal) | OV-RV | 0.407 | 0.012 | 0.048 |
|  |  | SKAT-O-C | 0.271 | 0.301 | 0.170 |
|  |  | SKAT-O | 0.048 | 0.147 | 0.063 |
|  |  | SKAT-C | 0.101 | 0.380 | 0.265 |
|  |  | SKAT | 0.016 | 0.202 | 0.112 |
|  |  | CAST | 0.117 | 0.003 | 0.007 |
|  |  | SUM | 0.114 | 0.007 | 0.003 |
|  |  | WSS | 0.056 | 0.008 | 0.003 |
|  | 40 (18 causal) | OV-RV | 0.638 | 0.095 | 0.013 |
|  |  | SKAT-O-C | 0.445 | 0.179 | 0.527 |
|  |  | SKAT-O | 0.082 | 0.027 | 0.264 |
|  |  | SKAT-C | 0.098 | 0.236 | 0.662 |
|  |  | SKAT | 0.001 | 0.047 | 0.368 |
|  |  | CAST | 0.226 | 0.011 | 0.029 |
|  |  | SUM | 0.178 | 0.007 | 0.022 |
|  |  | WSS | 0.113 | 0.004 | 0.055 |
| $\alpha=0.05$ | 20 (12 causal) | OV-RV | 0.664 | 0.063 | 0.178 |
|  |  | SKAT-O-C | 0.657 | 0.565 | 0.477 |
|  |  | SKAT-O | 0.209 | 0.323 | 0.215 |
|  |  | SKAT-C | 0.431 | 0.660 | 0.588 |
|  |  | SKAT | 0.097 | 0.430 | 0.317 |
|  |  | CAST | 0.230 | 0.020 | 0.032 |
|  |  | SUM | 0.205 | 0.017 | 0.022 |
|  |  | WSS | 0.231 | 0.023 | 0.027 |
|  | 40 (18 causal) | OV-RV | 0.840 | 0.227 | 0.070 |
|  |  | SKAT-O-C | 0.764 | 0.548 | 0.814 |
|  |  | SKAT-O | 0.299 | 0.144 | 0.511 |
|  |  | SKAT-C | 0.445 | 0.617 | 0.883 |
|  |  | SKAT | 0.010 | 0.213 | 0.652 |
|  |  | CAST | 0.365 | 0.035 | 0.142 |
|  |  | SUM | 0.428 | 0.047 | 0.055 |
|  |  | WSS | 0.387 | 0.039 | 0.15 |

**Table 2**: Power estimation of the TG gene in the presence of reverse causal variants

|  |  |  |  | $d=0.8$ |  |
| --- | --- | --- | --- | --- | --- |
| Level $\alpha$ | Number of RVs | Text | Pr=1/6 | Pr=1/3 | Pr=1/2 |
| $\alpha=0.01$ | 20 (12 causal) | OV-RV | 0.437 | 0.022 | 0.023 |
|  |  | SKAT-O-C | 0.320 | 0.138 | 0.543 |
|  |  | SKAT-O | 0.021 | 0.045 | 0.322 |
|  |  | SKAT-C | 0.203 | 0.223 | 0.648 |
|  |  | SKAT | 0.014 | 0.075 | 0.423 |
|  |  | CAST | 0.134 | 0.004 | 0.036 |
|  |  | SUM | 0.050 | 0.007 | 0.080 |
|  |  | WSS | 0.048 | 0.006 | 0.061 |
|  | 40 (18 causal) | OV-RV | 0.716 | 0.485 | 0.201 |
|  |  | SKAT-O-C | 0.534 | 0.376 | 0.761 |
|  |  | SKAT-O | 0.133 | 0.041 | 0.621 |
|  |  | SKAT-C | 0.245 | 0.326 | 0.809 |
|  |  | SKAT | 0.008 | 0.028 | 0.688 |
|  |  | CAST | 0.259 | 0.137 | 0.219 |
|  |  | SUM | 0.247 | 0.106 | 0.239 |
|  |  | WSS | 0.176 | 0.055 | 0.232 |
| $\alpha=0.05$ | 20 (12 causal) | OV-RV | 0.688 | 0.064 | 0.068 |
|  |  | SKAT-O-C | 0.703 | 0.390 | 0.791 |
|  |  | SKAT-O | 0.196 | 0.176 | 0.598 |
|  |  | SKAT-C | 0.605 | 0.534 | 0.867 |
|  |  | SKAT | 0.130 | 0.280 | 0.704 |
|  |  | CAST | 0.267 | 0.038 | 0.119 |
|  |  | SUM | 0.257 | 0.038 | 0.221 |
|  |  | WSS | 0.243 | 0.036 | 0.203 |
|  | 40 (18 causal) | OV-RV | 0.882 | 0.727 | 0.382 |
|  |  | SKAT-O-C | 0.821 | 0.734 | 0.888 |
|  |  | SKAT-O | 0.399 | 0.239 | 0.787 |
|  |  | SKAT-C | 0.625 | 0.704 | 0.925 |
|  |  | SKAT | 0.086 | 0.181 | 0.840 |
|  |  | CAST | 0.507 | 0.309 | 0.478 |
|  |  | SUM | 0.452 | 0.261 | 0.489 |
|  |  | WSS | 0.464 | 0.260 | 0.443 |

1. The numerical performance of the testing procedure based on the statistic $C-D$.

**Table 3**: Estimated power results of the eight methods based on the generated genotypes without the denominator of $\gamma$-statistic

| Level $\alpha$ | Number of RVs | Text | $d=0.8$ | $d=0.6$ | $d=0.4$ | $d=0.2$ |
| --- | --- | --- | --- | --- | --- | --- |
| $\alpha=0.01$ | 20 (12 causal) | OV-RV | 0.735 | 0.580 | 0.244 | 0.064 |
|  |  | SKAT-O-C | 0.667 | 0.477 | 0.147 | 0.031 |
|  |  | SKAT-O | 0.286 | 0.202 | 0.054 | 0.022 |
|  |  | SKAT-C | 0.213 | 0.062 | 0.012 | 0.006 |
|  |  | SKAT | 0.012 | 0.006 | 0.001 | 0.005 |
|  |  | CAST | 0.398 | 0.277 | 0.115 | 0.038 |
|  |  | SUM | 0.403 | 0.305 | 0.106 | 0.029 |
|  |  | WSS | 0.306 | 0.243 | 0.080 | 0.031 |
|  | 40 (18 causal) | OV-RV | 0.812 | 0.523 | 0.253 | 0.066 |
|  |  | SKAT-O-C | 0.776 | 0.368 | 0.173 | 0.035 |
|  |  | SKAT-O | 0.347 | 0.137 | 0.065 | 0.013 |
|  |  | SKAT-C | 0.224 | 0.041 | 0.014 | 0.004 |
|  |  | SKAT | 0.010 | 0.002 | 0.002 | 0.003 |
|  |  | CAST | 0.423 | 0.228 | 0.101 | 0.034 |
|  |  | SUM | 0.472 | 0.239 | 0.114 | 0.035 |
|  |  | WSS | 0.374 | 0.185 | 0.087 | 0.027 |
| $\alpha=0.05$ | 20 (12 causal) | OV-RV | 0.901 | 0.764 | 0.452 | 0.179 |
|  |  | SKAT-O-C | 0.865 | 0.713 | 0.347 | 0.117 |
|  |  | SKAT-O | 0.546 | 0.441 | 0.198 | 0.083 |
|  |  | SKAT-C | 0.512 | 0.249 | 0.070 | 0.045 |
|  |  | SKAT | 0.071 | 0.038 | 0.016 | 0.029 |
|  |  | CAST | 0.631 | 0.472 | 0.264 | 0.094 |
|  |  | SUM | 0.689 | 0.556 | 0.293 | 0.111 |
|  |  | WSS | 0.637 | 0.515 | 0.266 | 0.102 |
|  | 40 (18 causal) | OV-RV | 0.928 | 0.736 | 0.479 | 0.179 |
|  |  | SKAT-O-C | 0.916 | 0.670 | 0.416 | 0.127 |
|  |  | SKAT-O | 0.625 | 0.384 | 0.219 | 0.079 |
|  |  | SKAT-C | 0.532 | 0.236 | 0.088 | 0.029 |
|  |  | SKAT | 0.054 | 0.029 | 0.015 | 0.021 |
|  |  | CAST | 0.648 | 0.466 | 0.283 | 0.110 |
|  |  | SUM | 0.739 | 0.511 | 0.320 | 0.134 |
|  |  | WSS | 0.671 | 0.482 | 0.281 | 0.114 |

**Table 4**: Estimated power results of the TG gene of the six methods without the denominator of $\gamma$-statistic

| Level $\alpha$ | Number of RVs | Text | $d=0.8$ | $d=0.6$ | $d=0.4$ | $d=0.2$ |
| --- | --- | --- | --- | --- | --- | --- |
| $\alpha=0.01$ | 20 (12 causal) | OV-RV | 0.832 | 0.595 | 0.258 | 0.058 |
|  |  | SKAT-O-C | 0.684 | 0.392 | 0.122 | 0.022 |
|  |  | SKAT-O | 0.220 | 0.104 | 0.036 | 0.007 |
|  |  | CAST | 0.572 | 0.338 | 0.139 | 0.032 |
|  |  | SUM | 0.333 | 0.155 | 0.062 | 0.010 |
|  |  | WSS | 0.332 | 0.168 | 0.065 | 0.011 |
|  | 40 (18 causal) | OV-RV | 0.942 | 0.708 | 0.400 | 0.091 |
|  |  | SKAT-O-C | 0.868 | 0.577 | 0.241 | 0.034 |
|  |  | SKAT-O | 0.383 | 0.189 | 0.115 | 0.015 |
|  |  | CAST | 0.604 | 0.334 | 0.201 | 0.039 |
|  |  | SUM | 0.652 | 0.378 | 0.208 | 0.041 |
|  |  | WSS | 0.467 | 0.268 | 0.174 | 0.028 |
| $\alpha=0.05$ | 20 (12 causal) | OV-RV | 0.946 | 0.804 | 0.479 | 0.173 |
|  |  | SKAT-O-C | 0.887 | 0.671 | 0.338 | 0.098 |
|  |  | SKAT-O | 0.552 | 0.330 | 0.138 | 0.041 |
|  |  | CAST | 0.738 | 0.502 | 0.255 | 0.094 |
|  |  | SUM | 0.716 | 0.476 | 0.243 | 0.089 |
|  |  | WSS | 0.722 | 0.492 | 0.252 | 0.098 |
|  | 40 (18 causal) | OV-RV | 0.982 | 0.886 | 0.628 | 0.219 |
|  |  | SKAT-O-C | 0.977 | 0.824 | 0.507 | 0.142 |
|  |  | SKAT-O | 0.754 | 0.495 | 0.309 | 0.093 |
|  |  | CAST | 0.820 | 0.598 | 0.402 | 0.120 |
|  |  | SUM | 0.834 | 0.629 | 0.414 | 0.123 |
|  |  | WSS | 0.806 | 0.602 | 0.396 | 0.131 |
